# Mouse-tracking reveals cognitive conflict during negative impression formation in women with Borderline Personality Disorder or Social Anxiety Disorder

**DOI:** 10.1371/journal.pone.0247955

**Published:** 2021-03-04

**Authors:** Johanna Hepp, Pascal J. Kieslich, Andrea M. Wycoff, Katja Bertsch, Christian Schmahl, Inga Niedtfeld

**Affiliations:** 1 Department of Psychosomatic Medicine and Psychotherapy, Central Institute of Mental Health, Medical Faculty Mannheim, Heidelberg University, Heidelberg, Germany; 2 Department of Psychology & Mannheim Centre for European Social Research (MZES), School of Social Sciences, University of Mannheim, Mannheim, Germany; 3 Department of Psychological Sciences, University of Missouri, Columbia, MO, United States of America; 4 Department of Psychology, Ludwig-Maximilians-University Munich, Munich, Germany; Medical University of Vienna, AUSTRIA

## Abstract

Individuals with Borderline Personality Disorder (BPD) or Social Anxiety Disorder (SAD) suffer from substantial interpersonal dysfunction and have difficulties establishing social bonds. A tendency to form negative first impressions of others could contribute to this by way of reducing approach behavior. We tested whether women with BPD or SAD would show negative impression formation compared to healthy women (HCs). We employed the Thin Slices paradigm and showed videos of 52 authentic target participants to 32 women with BPD, 29 women with SAD, and 37 HCs. We asked participants to evaluate whether different positive or negative adjectives described targets and expected BPD raters to provide the most negative ratings, followed by SAD and HC. BPD and SAD raters both agreed with negative adjectives more often than HCs (e.g., ‘Yes, the person is greedy’), and BPD raters rejected positive adjectives more often (e.g., ‘No, the person is not humble.’). However, BPD and SAD raters did not differ significantly from each other. Additionally, we used the novel process tracing method *mouse-tracking* to assess the cognitive conflict (via trajectory deviations) raters experienced during decision-making. We hypothesized that HCs would experience more conflict when making unfavorable (versus favorable) evaluations and that this pattern would flip in BPD and SAD. We quantified cognitive conflict via maximum absolute deviations (MADs) of the mouse-trajectories. As hypothesized, HCs showed more conflict when rejecting versus agreeing with positive adjectives. The pattern did not flip in BPD and SAD but was substantially reduced, such that BPD and SAD showed similar levels of conflict when rejecting and agreeing with positive adjectives. Contrary to the hypothesis for BPD and SAD, all three groups experienced substantial conflict when agreeing with negative adjectives. We discuss therapeutic implications of the combined choice and mouse-tracking results.

## Introduction

The ability to correctly infer the characteristics and intentions of others is vital for adaptive social interaction [1]. Even brief, initial inferences about interaction partners, such as first impressions, can determine approach–avoidance tendencies and greatly influence the likelihood of establishing social bonds and the quality of interaction [2, 3]. Negative first impressions, in particular, tend to be stable and decrease the probability of future interactions [4]. Therefore, a tendency to form negative first impressions of others may help explain difficulties with establishing new relationships and interpersonal problems in individuals with psychopathology. Two types of psychopathology that are particularly (but in no way exclusively) characterized by interpersonal difficulties are Borderline Personality Disorder (BPD) and Social Anxiety Disorder (SAD). Individuals with BPD and SAD both suffer from high levels of loneliness, a low number of interpersonal relationships, and low relationship quality [5–9]. Both disorders also involve a negative processing of social cues that has been implied theoretically and demonstrated empirically [for reviews, see 10–13].

Theoretical models of BPD, such as the Biosocial Model [14, 15], posit that individuals with BPD show distorted social information processing under stress, often manifesting as a misinterpretation of social cues as threatening. This can include an interpretation of other individuals as threatening or negative in a broader sense. In addition, mentalization-based models of BPD suggest that BPD typically entails insecure or disorganized attachment styles, which are characterized by unstable and negative expectations of others [16, 17]. These negative expectations may not only apply to existing relationships (e.g. friends or family) but also extend to interactions with strangers and affect the formation of first impressions. Lastly, psychoanalytic models of BPD assume that BPD individuals have ‘split’ representations of others, such that they tend to represent others in extremes of all-good or, more often, all-bad [18]. It is likely that this dichotomized and negative mental representation of others might also contribute to the process of impression formation and result in negative distortions. In sum, each of the three big theoretical models of BPD implies that BPD individuals would form negative first impressions of others.

Theories of SAD make similar predictions. Cognitive behavioral models of SAD propose that individuals with SAD believe they are inferior and that others will ridicule them. Consequently, they scan their social surroundings for cues that others are rejecting or ridiculing them, are hyper vigilant toward even small indicators of potential rejection, and tend to choose negative interpretations even when benevolent interpretations are available (e.g. when the presented stimuli are neutral and would allow both positive and negative interpretations) [19, 20]. The tendency to scan for negative cues likely affects the process of impression formation for individuals with SAD as well and may also result in negative first impressions of others. In the present study, we aimed to test these predictions. Specifically, we used the *Thin Slices paradigm* [21] to assess whether individuals with BPD or SAD form negative first impressions of others.

The *Thin Slices paradigm* has been used to investigate impression formation in numerous previous studies [21]. In this paradigm, participants see a ‘thin slice’ of behavior, typically a photograph or short video sequence, of a target participant and are asked to evaluate the target’s personality. To date, there are three studies with BPD raters that employed this paradigm [22–24]. In all three studies, individuals with BPD tended to evaluate targets more negatively than healthy control groups, both in open and closed response formats. Some of the observed effects were also stronger in BPD compared to clinical control participants. Specifically, BPD participants evaluated targets more negatively on aggression-related traits than participants with depression and evaluated targets showing BPD relevant behavior (e.g., abandonment) more poorly than participants with a cluster C personality disorder. Adding to this, one study also assessed how BPD individuals perceive real-life interaction partners [25]. The authors found that participants with BPD (versus community controls) tended to perceive real-life interaction partners as more cold or quarrelsome, which then resulted in negative affect and cold-quarrelsome behavior on the side of the BPD individual. This study is particularly important because it demonstrates how evaluating others negatively can exacerbate symptoms of BPD.

Studies with SAD samples have not used the Thin Slices paradigm, but employed somewhat similar paradigms that might also shed light on whether individuals with SAD would evaluate strangers negatively. In one study, participants gave a speech while viewing a video of a crowd acting neutral, which participants knew was pre-recorded [26]. Results showed that higher levels of social anxiety were associated with significantly lower evaluations of crowd friendliness, pleasantness, and interest. In a similar study [27], highly socially anxious participants evaluated a crowd as less appreciative of their speech than low socially anxious participants. In a third study, socially anxious participants gave a speech that was observed by a confederate who showed ambiguous behavior. Participants with higher social anxiety evaluated the confederate as more threatening [28]. Although these studies highlight SAD individuals’ negative evaluations of strangers, two studies asked SAD individuals to evaluate crowds and not individual targets. Moreover, all studies used targets that did not show authentic behavior, as confederates and crowds were instructed by the experimenters. The studies also used paradigms that created a threatening scenario for participants in which they felt that they (and their speeches) would be evaluated and therefore had a specific motivation to be vigilant for potential negative cues. It has not yet been tested whether SAD individuals would show the same tendency to perceive others negatively outside of a threatening context.

### Mouse-tracking as a process-tracing measure

**Fig 1:**
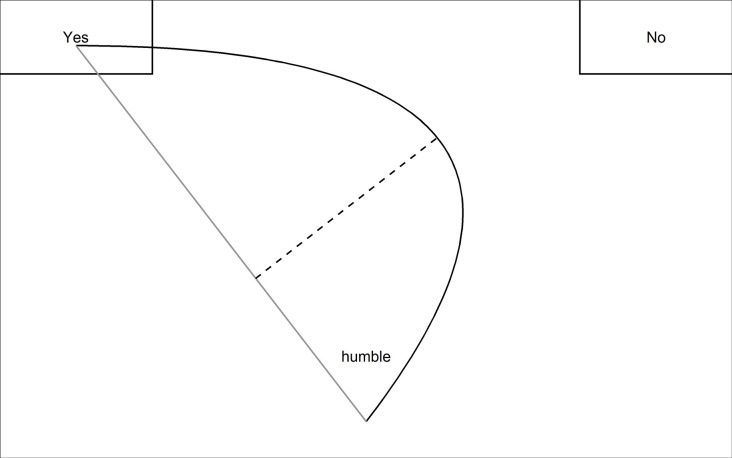
Exemplary mouse-tracking slide as included in the present study. The maximum absolute deviation is presented as a dotted line.

As detailed above, theories of BPD and SAD suggest that these individuals form negative first impressions of strangers [15, 18–20, 29], and empirical studies have generated some support for this hypothesis [22–28]. However, evidence on impression formation in BPD and SAD is based solely on the analysis of choice outcomes, such as ratings on numerical scales. What remains largely unknown is the underlying cognitive process of *how* participants decide that someone appears more positive or negative to them. A novel method that could provide insight into this process is *mouse-tracking*. This involves continuously tracking participants’ computer-mouse cursor movements while they complete a decision task. Specifically, both the spatial position on the screen and the time-point when that position is reached are logged. Thus, mouse-tracking data comprises both spatial and temporal information. Mouse-tracking paradigms usually include tasks that require a choice between two spatially separated response options. For instance, this could be a ‘yes’ and a ‘no’ button that are placed in the top left and top right corner of the screen (See Fig 1 for an illustration). Participants first have to click on a start button, usually placed in the bottom center of the screen, to start a trial. After this click, the stimulus is presented and the cursor movements are recorded until participants indicate their choice by clicking on one of the buttons.

The central assumption in mouse-tracking is that the relative cognitive activation of the two response options is reflected in the mouse movements [30, 31]. In other words, the more a participant considers a response option, the more their cursor moves toward it. If a participant is highly clear in their decision, they will move the cursor to one response option in a relatively straight line. If the decision creates cognitive conflict between the two response options, the cursor movement will likely show a pattern that is curved (that oscillates between both options), with greater curvature implying greater cognitive conflict. It is believed that the hand movements in mouse-tracking paradigms reflect neurological processes underlying the decision process [for an overview, see 30], for instance the preparation of multiple motor plans in the motor cortex [32]. The degree of cognitive conflict captured via mouse-movements has been associated with the level of anterior cingulate cortex activity [33], a brain region that is central to conflict detection and resolution [34, 35]. Mouse-tracking as a process tracing method was initially introduced in the area of language processing [36], but has since spread across a broad range of psychological fields [for recent reviews, see 37, 38]. Yet, except for a recent study that used mouse-tracking to detect faking behavior in a personality and psychopathology questionnaire in a non-clinical sample [39], mouse-tracking has (to the best of our knowledge) not been applied to clinical populations.

In the current study, we used mouse-tracking to assess the process of impression formation in BPD and SAD. Specifically, we aimed to measure the level of cognitive conflict experienced during impression formation, as this could hold important treatment implications. For instance, curvature toward a positive evaluation, even when the final choice is a negative one, would indicate that positive options are already being considered by patients. This existing activation of the positive option could be fostered therapeutically. In contrast, negative evaluations during which the positive option was barely considered (with straight lines to the negative evaluation) would imply that the positive option must first be proposed as a possibility.

### The present study

The aim of the present study was to assess whether individuals with BPD and SAD form more unfavorable first impressions of strangers shown to them in short video sequences (‘Thin Slices’) than healthy control participants (HCs). In addition to assessing impression formation at the choice level, we included mouse-tracking as a process tracing measure. Specifically, we recorded raters’ mouse movements while they evaluated targets on a range of positive and negative adjectives (e.g. humble, greedy). Raters decided whether each adjective applied to each target individual by moving their mouse cursor from a start button to a ‘yes’ or a ‘no’ button (see Fig 1).

### Choice-level hypotheses

For the choice level, we hypothesized that participants in the BPD group and the SAD group would rate targets more negatively than HCs. This hypothesis was derived from the aforementioned theoretical models of BPD, including the biosocial model [15], mentalization-based model [16], and object-relations model of BPD [18], as well as the cognitive model of SAD [19], which predict negative impression formation in BPD and SAD. Moreover, previous empirical studies using the Thin Slices paradigm in BPD samples [22–24] and similar paradigms in SAD samples support this notion [26–28].

In addition to expecting a difference between the two clinical groups and the HC group, we expected stronger effects in BPD compared to SAD. This was based on a crucial difference between theories of BPD and SAD: Theories of BPD suggest that negative representations of others are pervasive across contexts, whereas theories of SAD suggest that negative aspects of others primarily become relevant in evaluative situations, in which the SAD individual expects ridicule or rejection [19, 20]. In BPD, the tendency to perceive others negatively is assumed to be more generalized. Due to biographies and learning histories that are marked by adverse events and invalidating environments [14, 15], negative expectations of others in BPD tend to go beyond rejection, ridicule, and devaluation. Rather, they extend to expectations of abandonment, betrayal, unfairness, breaking of trust, and subjugation, thereby generalizing outside of evaluative contexts [15, 40]. Thus, we derived the following between-groups hypotheses, expecting an order of BPD>SAD>HC.

*H1a*: Compared to HCs, participants with BPD or SAD will more often reject positive adjectives (i.e., choose ‘no’ when positive adjectives are presented) and this effect will be stronger in BPD than in SAD.

*H1b*: Compared to HCs, participants with BPD or SAD will more often agree that negative adjectives describe targets (i.e., choose ‘yes’ when negative adjectives are presented) and this effect will be stronger in BPD than in SAD.

### Process-level hypotheses

In addition to our choice-level hypotheses, we had hypotheses about the process level that we aimed to test using mouse-tracking. These hypotheses pertained to the level of cognitive conflict experienced during impression formation. For healthy individuals, the person positivity bias suggests a general tendency to evaluate individuals favorably [41] and this was supported by previous Thin Slices studies using non-clinical samples [e.g., 42, 43]. Thus, favorable evaluations can be considered the default response of healthy individuals. Evaluating someone unfavorably deviates from this default and should therefore incur cognitive conflict. In our specific case, this implied that HC raters should experience more conflict when saying ‘no’ (vs. ‘yes’) to a positive adjective, and when saying ‘yes’ (vs. ‘no’) to a negative adjective.

In contrast to healthy individuals, those with BPD or SAD are unlikely to make favorable evaluations by default. Instead, theoretical models of BPD and SAD suggest they have cognitive schemas and lived experience (e.g., through trauma and bullying) of others being negative and should be biased toward unfavorable evaluations [14, 15, 18–20]. Therefore, BPD and SAD individuals should experience less (and not more) conflict when making unfavorable vs. favorable evaluations. We expected this effect to be stronger for BPD than for SAD, because cognitive schemas in SAD are largely restricted to others being a source of potential ridicule and rejection [19, 20], whereas in BPD they extend to themes of others being untrustworthy, dangerous, or even a source of abuse [15, 40]. In detail, we derived the following hypotheses:

*H2a*: HC raters will experience more cognitive conflict when making unfavorable than favorable evaluations.

*H2b*: BPD and SAD raters will differ from HC raters in that they experience less cognitive conflict when making unfavorable than favorable evaluations. This effect will be stronger in BPD than in SAD.

## Methods

### Participants

Ethics approval for this study was granted by the Medical Ethics Committee II of the medical faculty Mannheim at Heidelberg University (protocol no. 2013-654N-MA). Participants were recruited through the participant database of the clinical research unit KFO256 (www.kfo256.de) at the Central Institute of Mental Health in Mannheim, Germany, and through advertisements in online forums related to social anxiety and BPD. We recruited three groups of participants: Participants with a current BPD diagnosis, participants with a current SAD diagnosis, and healthy individuals without current or past psychopathology. Inclusion criteria for the BPD group were a current diagnosis of BPD according to the DSM-IV and no comorbid SAD. Inclusion criteria for the SAD group were a current SAD diagnosis according to DSM-IV and no more than three BPD criteria. General exclusion criteria were a current psychotic disorder, neurological dysfunction, severe head trauma, past-year substance dependence, and current psychotropic medication. All participants further completed a urine drug screening on the day of participation and were excluded from the sample if they had a positive screening. All participants were right-handed and used the computer-mouse with their right hand during the experiment.

Master’s level psychologists and trained clinicians working in the clinical research unit administered the German version of the Structured Clinical Interview for DSM-IV [SCID-I, 44] to determine SAD eligibility and comorbid diagnoses. To determine BPD diagnoses, interviewers further administered the International Personality Disorder Examination [IPDE, 45] and the Zanarini Rating Scale for Borderline Personality Disorder [ZAN-BPD, 46]. We report all diagnostic information for BPD and SAD participants in Table 1.

**Table 1:** Diagnostic data for the two clinical groups of participants with borderline personality disorder and social anxiety disorder.

	BPD		SAD	
	*N* = 32		*N* = 29	
	*N*	%	*N*	%
*Any anxiety disorder (other than SAD)*	14	43.75	5	17.24
Post-traumatic stress disorder	11	34.38	2	6.90
Panic disorder	6	18.75	3	10.34
Obsessive compulsive disorder	4	12.50	0	0
*Any mood disorder*	13	40.62	6	17.24
Current major depressive episode	12	37.50	4	13.79
Dysthymia	5	15.62	3	10.34
*Any eating disorder*	9	28.12	3	10.34
Bulimia nervosa	7	21.88	2	6.90
Eating disorder NOS	2	6.25	0	0
Anorexia nervosa	0	0	1	3.45
*Alcohol use disorder*	3	9.38	0	0

*Note*. BPD *=* Borderline Personality Disorder, SAD = Social Anxiety Disorder. Participants in the healthy control group (n = 37) had no current or past mental illnesses. Comorbid diagnoses were missing for 1 participant in the BPD group and 1 participant in the SAD group.

We recruited a total of 106 women, including 40 women with a current BPD diagnosis based on the IPDE interviews, 29 women with a current SAD diagnosis based on the SCID-I, and 37 healthy women without any current or lifetime diagnoses. One BPD participant was excluded from the sample because of a positive THC screening on the day of participation, and one additional BPD participant was excluded due to missing diagnostic details. Six BPD participants were excluded because they suffered from comorbid SAD. Participants were recruited for a larger research program within the clinical research unit KFO256 (www.kfo256.de) at the Central Institute of Mental Health in Mannheim, Germany. The overall research program did not have SAD comorbidity as an exclusion criterion; therefore, these participants were part of the initially recruited group of BPD participants and then were excluded for this specific experiment. Thus, the final dataset included 98 participants (32 BPD, 29 SAD, 37 HC). BPD participants had between 0 and 6 comorbid diagnoses (*M* = 1.94, *SD* = 1.63) and SAD participants had between 0 and 3 comorbid diagnoses (*M* = 0.82, *SD* = 1.02), which resulted in significantly less comorbidity for SAD than BPD participants (*t*(58) = 3.23, *p* = .002). The most common comorbid condition was a current mood disorder in both BPD participants (40.6%) and SAD participants (17.2%). Consequently, we statistically adjusted for any comorbid mood disorder in all analyses.

Age in the total sample ranged from 18 to 52, and mean ages did not differ significantly between groups (*M*_BPD_ = 29.22, *SD*_BPD_ = 6.80; *M*_SAD_ = 27.5, *SD*_SAD_ = 8.61, *M*_HC_ = 29.65, *SD*_HC_ = 9.92; BPD vs. SAD: *t*(58) = 0.86, *p* = .391; BPD vs. HC: *t*(67) = -0.21, *p* = .837, SAD vs. HC: *t*(63) = 0.92, *p* = .364). In all three groups, most participants were currently single, identified as heterosexual, had received at least 12 years of education (and therefore obtained a university entrance level degree in Germany), were currently studying or employed, and had a monthly income of less than 1,000 Euros. Detailed demographic information for each group is presented in Table 2. The current study is part of a larger study program and therefore we make note of a number of closely related manuscripts (see also the S1 Text section for more detail). In phase I of the study program, we created the Thin Slices target videos by filming participants with BPD and HCs. The target sample was first reported in [54]. We then used this set of target videos for a range of investigations. In phase II, we showed target videos to samples of student raters. We were interested whether targets with BPD would be perceived differently than HC targets and found that individuals with BPD were perceived more negatively than HCs [54]. Next, we asked a third sample of students to rate observable behavioral cues for all targets, e.g., amount of eye-contact with the camera, and used this as a mediator for the previously observed negative ratings [53]. Thus, all studies in phase II investigated BPD individuals as *targets*. The current study is part of phase III, in which we investigated BPD participants as *raters* of Thin Slices. We previously published data on other variables that were assessed within the present rater sample [48]. The previously published data included data from an economic game, trustworthiness, approachability, and similarity ratings (none of which were mouse-tracked). The previous manuscript addressed interaction effects between target and rater groups (e.g., whether BPD raters perceive BPD targets differently). Although the present and the previous study used the same stimulus material and rater sample, they report different sets of variables that do not overlap.

**Table 2:** Demographic data for the three rater groups.

	BPD (*N* = 32)		SAD (*N* = 29)		HC *(N* = 37)	
	*N*	%	*N*	%	*N*	%
*Monthly income*						
< 500 €	9	28.12	4	13.79	1	2.7
500–1000 €	10	31.25	15	51.72	16	43.24
1000–1500 €	6	18.75	7	24.14	9	24.32
1500–2000 €	5	15.62	2	6.90	6	16.22
> 2000 €	1	3.12	1	3.45	5	13.51
*Relationship status*						
Single	23	71.88	22	75.86	29	78.38
Married	3	9.38	5	17.54	7	18.92
Long-term relationship	3	9.38	0	0	0	0
Divorced	3	9.38	2	6.90	1	2.7
*Employment status*						
Student	8	25.0	17	58.62	18	48.65
Employed	11	34.38	7	24.14	16	43.24
Unemployed	7	21.88	3	10.34	3	8.11
Retired	2	6.25	0	0	0	0
Long-term sick leave	4	12.5	1	3.45	1	2.7
*Education*						
Graduation after 9 yrs	1	3.12	1	3.45	1	2.7
Graduation after 10 yrs	9	28.12	3	10.34	7	18.92
Graduation after 12–13 yrs	13	40.62	15	51.72	16	43.24
University degree	9	28.12	10	34.48	13	35.14
*Sexual orientation*						
Heterosexual	20	60.50	24	82.76	35	94.59
Homosexual	3	9.38	2	6.90	1	2.7
Bisexual	9	28.12	1	3.45	1	2.7

*Note*. Two participants did not indicate their sexual orientation.

### Design

Participants in the present study completed an initial phone screening and, if eligible, were then invited to an in-person clinical interview session. At this session, they received detailed information about the study procedure and provided written informed consent. Next, participants completed diagnostic interviews, filled out questionnaires, and provided demographic data. Following a short break, participants then completed the experimental part of the study in the laboratory. Participants completed the experiment with their dominant, right hand. Participants were paid 13 Euros per hour for their participation (including the diagnostic interview).

We created the Thin Slices experiment using the free, open-source experiment builder *OpenSesame* [47] and presented it on a computer screen with a resolution of 1680 x 1050 px. First, participants received detailed instructions and completed 8 practice trials. During the experiment, participants saw 52 videos of authentic target participants that briefly spoke about their personal preferences. Videos were presented in a randomised order. After each target video, raters first saw a slide asking them to evaluate target qualities on a Likert-type scale, during which mouse-movements were *not* tracked. Data on these questions are presented in a previous publication by our group, which has no overlap with the variables used in the present study [48]. After this slide, raters were presented with 8 decision screens involving mouse-tracking in a randomized order. English translations of all experimental slides can be accessed online (https://osf.io/tqbka/).

On each mouse-tracking screen, participants were asked to indicate whether they thought a presented adjective applied to the target participant by clicking a ‘yes’ or ‘no’ button. We presented the adjective after participants had clicked on a start button at the bottom center of the screen and we recorded the mouse-trajectory from this point to the answer options ‘yes’ (does apply) or ‘no’ (does not apply; see Fig 1). Cursor-movements on the screen were tracked every 10 ms using the free OpenSesame plugin *mousetrap* [for further details, see 49 and material section]. Following recommendations in the mouse-tracking literature [50], mouse cursor acceleration was disabled and cursor speed was reduced. Clicking on the start button at the bottom centre of the screen served to align the start position of the mouse across trials. After the click, the cursor position was reset to the centre of the start button, the stimulus adjective was presented in the bottom centre of the screen, and cursor movements were recorded until participants clicked on one of the response buttons. For each target, adjectives were presented in a randomised order. The position of the yes and no response alternative (i.e., whether they were presented on the top left or right side of the screen) was counter balanced.

Based on the recorded cursor trajectory coordinates over time, there are different ways to quantify activation and response conflict [see 51 for an overview]. If one is interested in quantifying the maximum activation of the non-chosen option as an indicator of the maximum conflict that occurred in a specific trial, a simple and frequently used measure is the maximum absolute deviation (MAD) of the trajectory from an idealized straight line (see Fig 1). At present, MADs are the most commonly used measure to quantify cognitive conflict in mouse-tracking settings and were thus used in the current study. However, as it has recently been argued that mouse-tracking studies should go beyond analyzing trial summary statistics like MADs and instead take into account the complete shape of the trajectory [52], we also used the classified trajectory type instead of MADs as an outcome and present these results, along with an explanation of the method, in the S1 Text.

### Material

#### Video material

The Thin Slices video material used here was created by our group [53, 54]. Videos showed 52 target participants that spoke about their favorite meal, color, hobby, book, movie, animal, car, and holiday destination to give an impression of their personality. Target participants included 26 individuals with BPD that were recruited from the inpatient and outpatient unit at the Central Institute for Mental Health in Mannheim, Germany, and 26 healthy individuals that were recruited through our participant database. Healthy target participants were diagnosed by trained Master’s level psychologists and target patients were diagnosed by licensed and trained clinicians working in the outpatient unit or within the clinical research group. Axis-I diagnoses were assessed using the German version of the structured clinical interview for DSM-IV [SCID-I; 44], and personality disorders were assessed with the German adaptation of the International Personality Disorder Examination [IPDE; 45]. Both groups included 46% men and were matched for age and education. Further demographic and diagnostic details for the target participants are presented in Table 3. The target groups included individuals with BPD and HCs because, in two previous studies, we assessed whether BPD and HC individuals in the videos would be evaluated differently by samples of students [53, 54]. Thus, in the two previous studies, we assessed differences between the target groups, whereas in the current study, we were interested in differences between rater groups. Videos were cut to approximately 30 seconds in length and edited to have the same brightness, contrast, audio volume, and image section in each frame.

**Table 3:** Demographic data for target participants (presented in the Thin Slices videos) by group.

	BPD (*n* = 26)		HC (*n* = 26)
*Income per month*^*a*^			
< 1000 €	18 (69.2%)		10 (38.5%)
1000–2000 €	2 (7.7%)		6 (23.1%)
2000–3000 €	2 (7.7%)		6 (23.1%)
> 3000 €	0 (0.0%)		3 (11.5%)
*Relationship status*			
Single/ never married	16 (61.5%)		15 (57.7%)
Married/long-term relationship	6 (23.1%)		8 (30.7%)
Divorced	3 (11.5%)		3 (11.5%)
Widowed	1 (3.8%)		0 (0.0%)
*Occupation*			
Employed	10 (38.5%)		15 (57.7%)
Student	4 (15.4%)		11 (42.3%)
Unemployed	9 (34.6%)		0 (0.0%)
On pension	3 (11.5%)		0 (0.0%)
*Education*			
Without graduation	2 (7.7%)		0 (0.0%)
Graduation after 9 yrs	3 (11.5%)		2 (7.7%)
Graduation after 10 yrs	12 (46.2%)		7 (26.9%)
Graduation after 12–13 yrs	7 (26.9%)		6 (23.1%)
University degree	2 (7.7%)		11 (42.3%)

*Note*. BPD = Borderline Personality Disorder, HC = healthy control participants. Graduation after 9 years = “Hauptschulabschluss” in Germany, graduation after 10 years = “Realschulabschluss”, graduation after 12–13 years = “Abitur”. ^*a*^Four participants in the BPD group and one participant in the HC group did not provide data on their monthly income.

#### Mouse-tracking software

To record and analyze participants’ mouse-movements, we used the freely available software *mousetrap*. Mousetrap has two components. First, it provides a plugin that adds mouse-tracking functionality to the open source experiment builder OpenSesame [47] by extending its graphical user interface [49]. The mousetrap plugin is accompanied by the mousetrap R package that can be used to process and visualize the recorded mouse movement data and to calculate mouse-tracking indices for statistical analysis [51].

#### Adjectives

On each mouse-tracking slide, we presented one of eight adjectives. Six of these were based on the Honesty-Humility dimension of the HEXACO personality inventory, which measures dispositional cooperativeness [55]. The HEXACO model of personality is well-validated and the Honesty-Humility dimension is strongly related to prosocial behavior [56] as well as antisocial tendencies related to personality pathology [57]. We chose the Honesty-Humility dimension because those with BPD generally regard others as untrustworthy [58] and expect to be betrayed and abandoned by others [59]. The adjectives related to the Honesty-Humility dimension were evaluated in different languages [60, 61] and used in numerous studies, e.g., predicting antisocial behavior in the workplace [62]. The specific adjectives we used were *sincere*, *humble*, *just* (positive valence), and *greedy*, *hypocritical*, *malicious* (negative valence). We chose these adjectives because we considered them particularly relevant for interpersonal behaviour. We also included two adjectives that we deemed relevant to approach behaviour and that were included in previous Thin Slices studies [e.g., 63]: *likeable* and *interesting*.

### Data analysis

Data and analysis code are provided in an OSF online repository https://osf.io/tqbka/. We analyzed data with mixed effects models in R [64], using the *lmer* and *glmer* functions from the *lme4* package [65]. P-values were computed using the *lmerTest* package [66]. We employed mixed effects models to account for the crossed data structure and included crossed random intercepts for each rater and target. We excluded three participants from the analyses (2 BPD, 1 HC), because their mouse cursor and acceleration systems settings were accidentally left at the system default and hence differed from the other participants.

## Results

### Hypothesis 1

To test hypothesis 1 that BPD and SAD raters would evaluate targets more unfavorably than HC raters and that this effect would be stronger for BPD than for SAD, we employed a logistic mixed effects model treating the type of target evaluation as an outcome. That is, the outcome coded whether participants made an unfavorable evaluation (i.e., said ‘no’ to a positive adjective or said ‘yes’ to a negative adjective). This variable was coded 1 for unfavorable evaluations and 0 for favorable evaluations. The model included a dummy-coded predictor for adjective valence and a dummy-coded predictor for rater groups.

To facilitate interpretation, we ran the same model with three different combinations of coding the rater groups. First, we ran the model coding HC raters as baseline. This model version included a BPD dummy (BPD = 1, else = 0) and a SAD dummy (SAD = 1, else = 0). Thus, participants with values of 0 on both the BPD and SAD dummy were in the HC group. Because the main effects of the other predictors are interpretable only for the baseline rater group, we repeated the model coding BPD and SAD as baseline. In the BPD baseline model, we included a HC dummy (HC = 1, else = 0) and a SAD dummy (SAD = 1, else = 0). Finally, in the SAD baseline model, we included a HC dummy (HC = 1, else = 0) and a BPD dummy (BPD = 1, else = 0).

**Fig 2:**
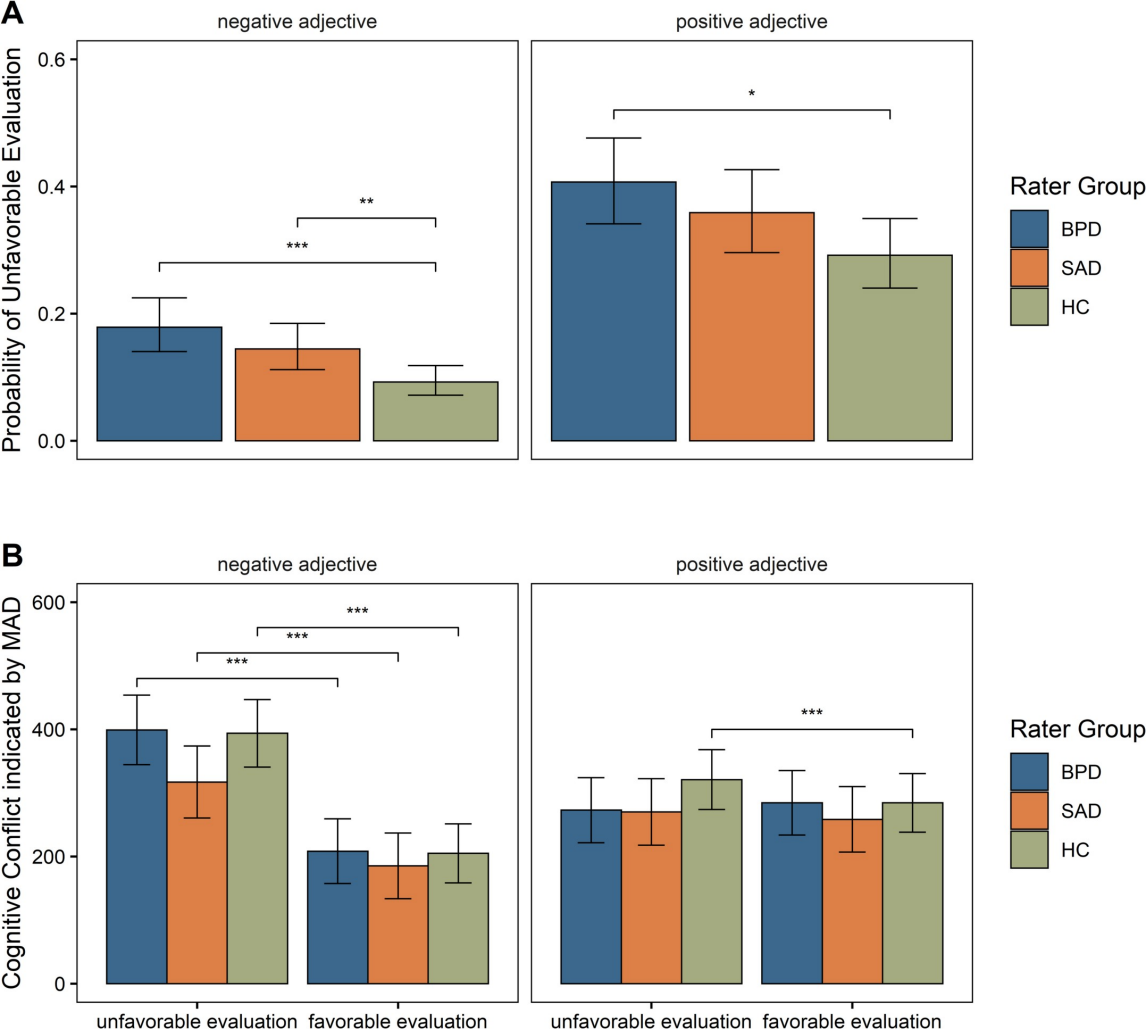
Illustration of results for hypotheses 1 and 2. Panel A presents the central results for hypothesis 1, depicting the probability of an unfavorable decision made by the three rater groups (BPD, SAD, HC), depending on whether a positive or negative adjective was presented. Panel B presents the central results for hypothesis 2, depicting cognitive conflict exhibited by the three rater groups, measured via maximum absolute deviation (MAD) in pixels, and depending on whether a positive or negative adjective was presented. Asterisks indicate significant effects at p < .05 *, p < .01 **, p < .001***.

Moreover, we coded adjective valence once using negative adjectives as the baseline condition and once using positive adjectives as the baseline condition. Results from all baseline combinations are presented in Table 4 and illustrated in Fig 2, panel A. The model further adjusted for any current depressive disorder covariate (DD), which we included because any current depressive disorder was the most common comorbid condition in both clinical groups. The DD covariate was coded 1 if a participant suffered from current depression or dysthymia and 0 if not.

**Table 4:** Results from a logistic mixed effects model with the probability of an unfavorable target evaluation as outcome, predicted by diagnostic group and adjective valence.

	Positive Adjective Baseline					Negative Adjective Baseline				
*Predictor*	*Est*.	*OR*	*95% CI*	*SE*	*p*	*Est*.	*OR*	*95% CI*	*SE*	*p*
	*HC Rater Baseline*									
Intercept	-0.89	0.41	[0.32; 0.54]	0.14	< .001	-2.29	0.10	[0.08; 0.13]	0.14	< .001
Adj. valence	**-1.40**	**0.25**	**[0.22; 0.27]**	**0.05**	**< .001**	**1.40**	**4.05**	**[3.68; 4.46]**	**0.05**	**< .001**
BPD (vs. HC)	**0.44**	**1.55**	**[1.09; 2.21]**	**0.18**	**.015**	**0.69**	**1.99**	**[1.38; 2.87]**	**0.19**	**< .001**
SAD (vs. HC)	0.27	1.31	[0.95; 1.82]	0.17	.105	**0.47**	**1.61**	**[1.14; 2.26]**	**0.17**	**.007**
DD Covariate	0.16	1.18	[0.82; 1.69]	0.19	.385	0.16	1.18	[0.82; 1.69]	0.19	.385
Adj. valence × BPD	**0.25**	**1.28**	**[1.12; 1.46]**	**0.07**	**< .001**	**-0.25**	**0.78**	**[0.68; 0.89]**	**0.07**	**< .001**
Adj. valence × SAD	**0.20**	**1.22**	**[1.07; 1.40]**	**0.07**	**.004**	**-0.20**	**0.82**	**[0.71; 0.94]**	**0.07**	**.004**
	*BPD Rater Baseline*									
Intercept	-0.45	0.64	[0.46; 0.88]	0.16	.007	-1.60	0.20	[0.15; 0.28]	0.17	< .001
Adj. valence	**-1.15**	**0.32**	**[0.29; 0.35]**	**0.05**	**< .001**	**1.15**	**3.16**	**[2.88; 3.45]**	**0.05**	**< .001**
HC (vs. BPD)	**-0.44**	**0.64**	**[0.45; 0.92]**	**0.18**	**.015**	**-0.69**	**0.50**	**[0.35; 0.72]**	**0.19**	**< .001**
SAD (vs. BPD)	-0.17	0.85	[0.60; 1.19]	0.18	.338	-0.22	0.81	[0.57; 1.15]	0.18	.232
DD Covariate	0.16	1.18	[0.82; 1.69]	0.19	.385	0.16	1.18	[0.82; 1.69]	0.19	.384
Adj. valence × HC	**-0.25**	**0.78**	**[0.68; 0.89]**	**0.07**	**< .001**	**0.25**	**1.28**	**[1.12; 1.46]**	**0.07**	**< .001**
Adj. valence × SAD	-0.05	0.95	[0.84; 1.09]	0.07	.480	0.05	1.05	[0.92; 1.20]	0.07	.480
	*SAD Rater Baseline*									
Intercept	-0.61	0.54	[0.40; 0.73]	0.15	< .001	-1.81	0.16	[0.12; 0.22]	0.15	< .001
Adj. valence	**-1.20**	**0.30**	**[0.27; 0.33]**	**0.05**	**< .001**	**1.20**	**3.31**	**[3.00; 3.64]**	**0.05**	**< .001**
HC (vs. SAD)	-0.27	0.76	[0.55; 1.06]	0.17	.105	**-0.47**	**0.62**	**[0.44; 0.88]**	**0.17**	**.007**
BPD (vs. SAD)	0.17	1.18	[0.84; 1.67]	0.18	.337	0.22	1.24	[0.87; 1.77]	0.18	.233
DD Covariate	0.16	1.18	[0.82; 1.69]	0.19	.385	0.16	1.18	[0.82; 1.69]	0.19	.385
Adj. valence × HC	**-0.20**	**0.82**	**[0.71; 0.94]**	**0.07**	**.004**	**0.20**	**1.22**	**[1.07; 1.40]**	**0.07**	**.004**
Adj. valence × BPD	0.05	1.05	[0.92; 1.29]	0.07	.480	-0.05	0.95	[0.84; 1.09]	0.07	.481

*Note*. Est. = estimate, OR = odds ratio, BPD = borderline personality disorder, SAD = social anxiety disorder, HC = healthy control participants, DD = current major depressive episode or dysthymia. Adjective valence was dummy-coded. Results for positive adjectives coded as baseline presented in the ‘positive adjectives’ column, and results for negative adjectives coded as baseline, presented in the negative adjectives column.

To assess the hypothesis that BPD and SAD raters would evaluate targets more unfavorably than HC raters, consider the upper panel of Table 4, which presents the results for the HC rater baseline. Here, the BPD dummy was significant and positive, indicating that BPD raters were more likely than HCs to evaluate targets unfavorably, by both agreeing with negative and rejecting positive adjectives more often. In contrast, the effect for the SAD group was only significant for negative adjectives. Thus, SAD raters evaluated targets more unfavorably than HC raters only by agreeing more with negative adjectives but not by rejecting positive adjectives more often.

In addition to a difference between both clinical groups and HCs, we hypothesized that BPD raters would show stronger tendencies for an unfavorable target evaluation than SAD raters. However, there was no significant difference between BPD and SAD raters. This was indicated by the non-significant effect of the SAD dummy in the BPD baseline (middle panel of Table 1) and the equivalent, non-significant effect of the BPD dummy in the SAD baseline (lower panel of Table 1). Thus, hypothesis 1 was partially supported, showing that BPD and SAD tended to make more unfavorable decisions than HC raters (though for SAD only by agreeing with negative adjectives), but that the difference between BPD and SAD was not significant.

The main effect for adjective valence was significant in all baseline rater combinations, indicating that all raters were more likely to evaluate targets unfavorably by rejecting positive adjectives. In other words, raters were more likely to make an unfavorable evaluation by saying ‘no’ to a positive adjective (e.g., “No, the person is *not* humble”) than by saying ‘yes’ to a negative adjective and actively ascribing targets a negative quality (e.g., “Yes, the person *is* greedy”). The effect of adjective valence was strongest in the HC group, followed by the SAD and the BPD groups. The two-way interactions between the rater groups and adjective valence demonstrated that the difference in the size of the effect of adjective valance was significant between the HC and BPD and between HC and SAD, but not between BPD and SAD. The odds ratios indicated that HC raters were 4.05 times more likely to evaluate a target unfavorably by rejecting a positive adjective than by agreeing with a negative adjective. SAD raters were 3.31 times more likely to make unfavorable judgments by disagreeing with positive adjectives, and BPD raters were 3.16 times. Thus, all raters tended to disagree with negative adjectives, but this was less pronounced for the clinical rater groups.

### Hypothesis 2

We hypothesized that HC raters would experience more cognitive conflict during unfavorable than during favorable evaluations and that this pattern would flip for BPD and SAD raters, such that they experience less cognitive conflict during unfavorable than during favorable evaluations. Importantly, we expected this effect to be stronger in BPD than in SAD. To assess level of cognitive conflict, we first computed MAD values for each trial using built-in functions from the R package *mousetrap* [51]. MAD values indicate how strongly participants deviate toward the unselected alternative in a specific trial and serve as a measure for the amount of cognitive conflict experienced in each trial, with greater values indicating more conflict. MAD values served as outcomes in two linear mixed effects models. In addition to these MAD-based models, we used trajectory prototypes as outcomes and all results replicated in this framework. Detailed results and an explanation of the prototype approach are presented in Table A1 in S1 Appendix.

**Fig 3:**
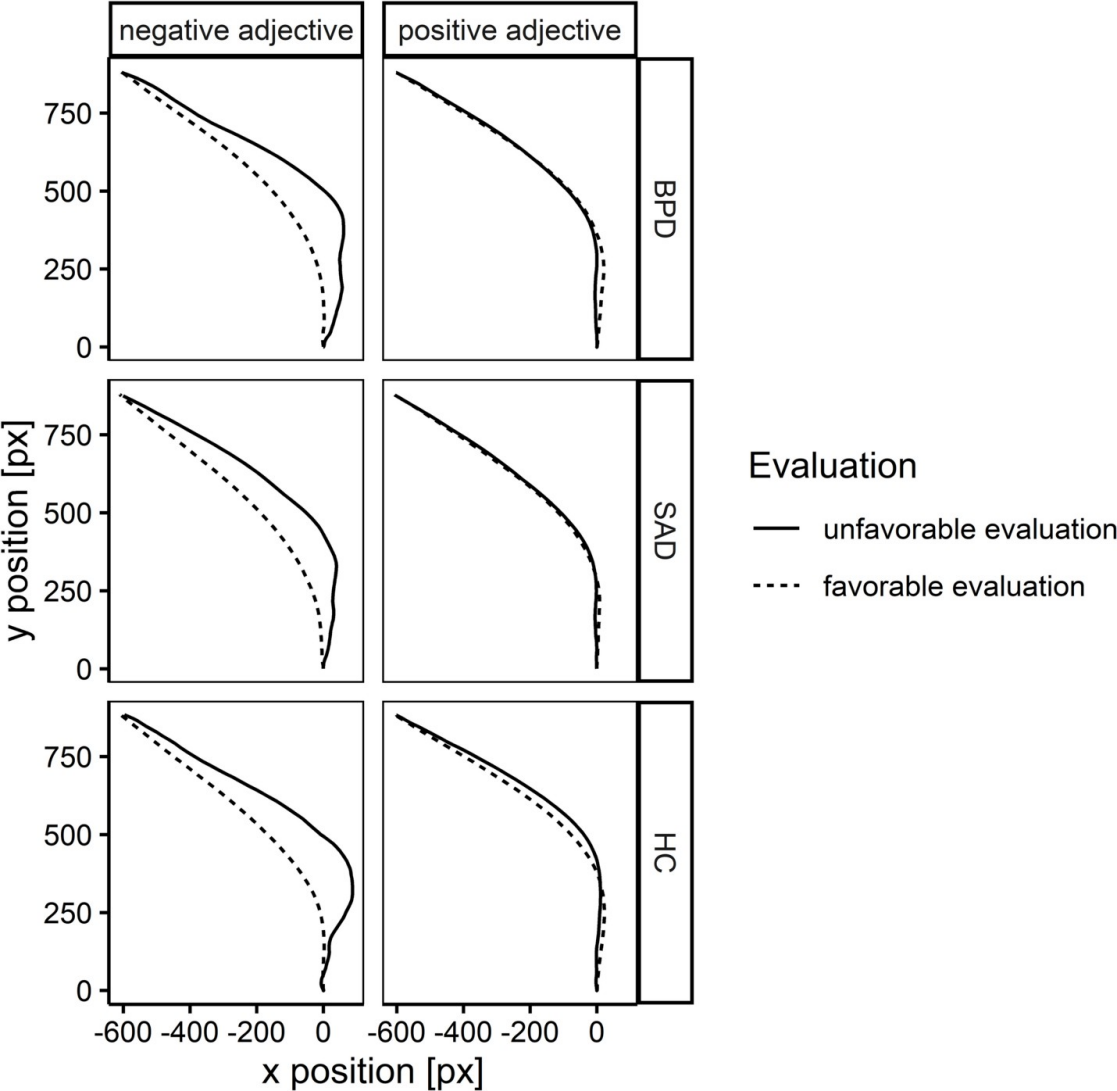
Illustration of aggregate mouse-trajectories. Trajectories presented for favorable and unfavorable target evaluations, separately for each rater group (BPD, SAD, HC), and depending on whether a negative or positive adjective was presented. All individual trajectories were flipped to the left, time-normalized into 101 bins, and aggregated separately per condition first within and then across raters.

We ran two separate models using MADs as outcomes, one for trials with positive adjectives and one for trials with negative adjectives. The models included an effect-coded predictor variable, indicating whether the rater’s target evaluation was unfavorable (coded 0.5) or favorable (coded -0.5). The models further included dummy variables for the three rater groups, and we modelled the interaction between the rater groups and the evaluation-type predictor. We again ran the model using all three baseline combinations, treating HC, BPD, and SAD as the rater baseline in the three model versions respectively. The model also included the DD covariate. The results are presented in Table 5 and illustrated in panel B of Fig 2. The aggregate mouse-trajectories for each rater group are presented in Fig 3.

**Table 5:** Results from two linear mixed effects models with MADs from mouse-tracking trials as outcome, predicted by diagnostic group and decision type; presented separately for negative and positive adjectives and for each of the rater group baselines.

	*Positive adjectives*			*Negative adjectives*		
*Predictor*	*Est*.	*SE*	*p*	*Est*.	*SE*	*p*
	*HC Rater Baseline*					
Intercept	302.86	24.64	< .001	293.74	23.75	< .001
Unfavorable evaluation	**36.65**	**8.49**	**< .001**	**175.16**	**14.74**	**< .001**
BPD (vs. HC)	-32.29	40.70	.430	-5.63	38.82	.885
SAD (vs. HC)	-42.82	37.73	.259	-50.03	36.14	.169
DD covariate	18.30	42.20	.666	33.13	39.64	.406
Unfavorable evaluation×BPD	**-46.49**	**12.35**	**< .001**	10.92	19.53	.576
Unfavorable evaluation×SAD	**-27.10**	**12.54**	**.031**	**-46.05**	**20.33**	**.024**
	*BPD Rater Baseline*					
Intercept	270.57	32.57	< .001	288.11	30.81	< .001
Unfavorable evaluation	-9.84	9.04	.277	**186.08**	**12.88**	**< .001**
HC (vs. BPD)	32.29	40.70	.430	5.63	38.82	.885
SAD (vs. BPD)	-10.53	39.47	.790	-44.40	37.54	.240
DD covariate	18.30	42.20	.666	33.13	39.64	.406
Unfavorable evaluation×HC	**46.49**	**12.35**	**< .001**	-10.92	19.53	.576
Unfavorable evaluation×SAD	19.40	12.94	.134	**-56.97**	**19.04**	**.003**
	*SAD Rater Baseline*					
Intercept	260.04	28.76	< .001	243.71	27.35	< .001
Unfavorable evaluation	9.56	9.34	.306	**129.11**	**14.07**	**< .001**
HC (vs. SAD)	42.82	37.73	.259	50.03	36.14	.169
BPD (vs. SAD)	10.53	39.47	.790	44.40	37.54	.240
DD covariate	18.30	42.20	.666	33.13	39.64	.406
Unfavorable evaluation×HC	**27.10**	**12.54**	**.031**	**46.05**	**20.33**	**.024**
Unfavorable evaluation×BPD	-19.40	12.94	.134	**56.97**	**19.04**	**.003**

*Note*. BPD = borderline personality disorder, SAD = social anxiety disorder / anxious avoidant personality disorder, HC = healthy control participants, DD = current major depressive episode or dysthymia. Unfavorable evaluation coded 0.5 when raters evaluated targets unfavorably and -0.5 when they evaluated targets favorably. Significant effects are highlighted in boldface.

In the first model, we only analyzed trials in which positive adjectives were presented to test whether HC raters would experience more conflict and whether BPD and SAD raters would experience less conflict when rejecting than agreeing with positive adjectives. As indicated by the significant main effect of ‘unfavorable evaluation’ in the upper left panel of Table 5, HC raters experienced more cognitive conflict when they evaluated targets unfavorably (i.e., by saying “no” to a positive adjective) than when they evaluated them favorably (i.e., by saying “yes” to a positive adjective). This finding supported H2a.

As shown in the middle and lower left table panels of Table 5 (main effect of ‘unfavorable evaluation’), the amount of conflict did not differ between unfavorable and favorable evaluations for BPD and SAD raters. Thus, contrary to H2b, the pattern observed in the HC group did not flip in the BPD and SAD groups. BPD and SAD raters did not experience less cognitive conflict when rejecting (versus agreeing with) positive adjectives, but they also did not experience more conflict, as HCs did.

In sum, for positive adjectives, HC raters showed a pattern of more conflict during unfavorable vs. favorable evaluations while BPD and SAD raters both showed a pattern of equal conflict during unfavorable and favorable evaluations. To determine whether these patterns differ significantly between the groups, consider the interaction terms ‘unfavorable evaluation×BPD’ and ‘unfavorable evaluation×SAD’ (upper left panel). The finding that these terms were both significant indicates that the BPD and SAD pattern differed significantly from the HC pattern. However, the BPD and SAD patterns did not differ significantly from each other, as indicated by the non-significant ‘unfavorable evaluation×SAD’ interaction term in the BPD baseline (middle panel).

In the second model, we only analyzed trials with negative adjectives to test whether HC raters would experience more conflict and whether BPD and SAD raters would experience less conflict when agreeing with versus rejecting negative adjectives. The results for the analyses using negative adjectives are presented in the right-hand columns of Table 5, separately for the three rater baseline combinations.

There was a significant main effect of ‘unfavorable evaluation’ for HC raters (upper panel), indicating that HC raters experienced more cognitive conflict when agreeing with negative adjectives (‘yes greedy’) than when rejecting negative adjectives (‘not greedy’). This supported H2a. Contrary to H2b, this effect did not flip in the BPD and SAD groups such that unfavorable evaluations entailed less conflict. Instead, both clinical groups also experienced *more* conflict when agreeing with vs. rejecting negative adjectives. This was indicated by the significant main effect of ‘unfavorable evaluation’ for the BPD rater baseline (middle panel) and the SAD rater baseline (lower panel). Thus, all three groups showed a pattern of more conflict when agreeing with versus rejecting negative adjectives. However, the difference in conflict between unfavorable and favorable evaluations was reduced in the SAD group compared to the HC group, as indicated by the significant ‘unfavorable evaluation×SAD’ interaction (in the HC baseline, upper panel). No significant differences emerged for this pattern when comparing the BPD group to either the SAD or HC group.

## Discussion

The present study assessed the cognitive process underlying impression formation in BPD and SAD. Specifically, we investigated whether individuals with BPD or SAD evaluate strangers more unfavorably, and whether they experience less cognitive conflict than HCs when doing so. We showed 52 short video sequences of target participants to 95 women with BPD or SAD and HCs. We presented participants with positive and negative adjectives (e.g., humble, greedy) and asked them to decide whether these adjectives applied to targets or not. During these trials, we recorded participants’ mouse movements to measure the amount of cognitive conflict they experienced during the decision process. Mouse-tracking is already widely applied in other psychological disciplines [37, 38] but, to the best of our knowledge, the current study marks the first application of mouse-tracking in a clinical sample.

First, we analyzed choice-level results and found support for negative impression formation in BPD and SAD (H1). We observed that both BPD and SAD raters evaluated targets unfavorably more often than HCs did, although for SAD raters this was only true with regard to a greater tendency to agree with negative adjectives. Descriptively, participant groups showed the expected order, such that BPD individuals made more unfavorable evaluations than SAD individuals, followed by HCs. Contrary to hypotheses, the BPD and the SAD groups did not differ significantly from each other. Including adjective type (positive vs. negative) in the model revealed that both BPD and SAD raters were more likely to agree with negative adjectives (e.g., ‘is greedy’) than HC raters, supporting H1a. Yet, only BPD raters were also more likely to reject positive adjectives (e.g., ‘not humble’) than HC raters, partially supporting H1b.

When participants evaluated targets negatively, they tended to do so by ‘saying no’ to positive adjectives more often than ‘saying yes’ to negative adjectives. Even BPD and SAD raters did not often ascribe negative qualities to targets. Even though their tendency was higher than that for HCs, BPD and SAD raters chose negative attributes in less than 20% of cases. Clinically, this implies that individuals with BPD or SAD are not necessarily hyper-focusing on negative attributes in others. Rather, they might be less ready to ascribe positive attributes to other people. Another potential explanation for this general tendency to avoid negative responses across all groups could be the presence of a socially desirable responding bias [67], which could result in participants avoiding rejecting positive attributes and ascribing negative ones.

The findings that both BPD and SAD participants tended to evaluate targets more unfavorably than HCs support theoretical models of both BPD [15, 16, 18] and SAD [19, 20]. Moreover, the present study adds to an existing body of evidence on negative impression formation in the Thin Slices paradigm for the BPD population [22–24]. Together with the findings depicted in our companion paper [48], the present study is the first application of the Thin Slices paradigm to a SAD sample, demonstrating that SAD individuals show a stronger tendency to ascribe negative attributes to targets than HCs. While focusing on authentic, individual targets and not crowds, our findings parallel previous evidence that highly socially anxious individuals tend to evaluate videos of crowds more negatively than low socially anxious individuals [26, 27].

In addition to analyzing choice-level data, we aimed to quantify the amount of cognitive conflict experienced during the decision process by analyzing participants’ computer mouse movements. By tracking participants’ mouse-cursor movements, we aimed to measure relative activation of the different response options presented on the mouse-tracking slides. That is, we measured how strongly raters tended toward the ‘yes’ and ‘no’ response options for each positive and negative adjective. We analyzed MAD values as an index for the maximum attraction to the non-chosen option (i.e., cognitive conflict) and replicated all analyses using the trajectory prototype approach (see S1 Appendix). We expected to see BPD and SAD raters experience less conflict when making unfavorable versus favorable evaluations, but this was not the case. Both clinical groups experienced similar levels of conflict when agreeing with positive adjectives as when rejecting them. Moreover, both clinical groups experienced *more* conflict when agreeing with negative adjectives than when rejecting them, which was contrary to our hypotheses.

As Fig 3 shows, especially when saying ‘yes’ to a negative adjective, the ‘no’ option exerted considerable pull in all groups. This suggests that BPD and SAD raters strongly considered favorable evaluations during the decision process, but ultimately discarded the favorable option more often than HC raters, which resulted in a higher number of unfavorable evaluations. This implies that the higher frequency of unfavorable evaluations by BPD and SAD raters was likely not an intuitive or automatic response made without a consideration of a favorable evaluation in the process. Rather, unfavorable evaluations seem to be the outcome of a process in which the favorable option was considered and then discarded. Clinically, this suggests that BPD and SAD individuals already do consider the favorable evaluation, highlighting the decision-making process as a possible target for cognitive intervention.

In sum, our findings add new insights to the body of work on impression formation in BPD and SAD. In addition to applying a new measure of cognitive conflict, the inclusion of both positive and negative adjectives alone allowed further insight into the process of impression formation. Our results suggest that negative impression formation in BPD and SAD is driven more strongly by a tendency to *not* ascribe positive attributes to targets, rather than to ascribe negative attributes to them. Importantly, the process of rejecting positive attributes was not accompanied by increased cognitive conflict in either clinical group, whereas for HC raters it was. At the same time, both clinical groups agreed with negative adjectives more often than HC raters, but this was accompanied by substantial conflict and contributed to only a small number of unfavorable evaluations. Most often, unfavorable evaluations were based on rejecting positive adjectives rather than agreeing with negative adjectives. In sum, evidence from choices and mouse-tracking suggests that those with BPD and SAD do not generally see targets more negatively but, rather, less positively.

### Limitations

The present study had a number of limitations. The first pertains to characteristics of the rater sample we recruited. BPD raters had a higher number of comorbid diagnoses than SAD raters. This problem is often encountered in BPD research due to an actual epidemiological phenomenon of higher comorbidity and lower functioning in BPD than in most other patient groups [e.g., 68]. Nevertheless, it also reduces the comparability of the two clinical groups we recruited. We provide trial level, descriptive, and demographic data online for readers that may be interested in further questions regarding comorbid conditions. Beyond issues pertaining to clinical characteristics, another limitation incurred by our sampling strategy is gender selectivity, as we only recruited women for the rater sample. This restricts the generalizability of the current findings to women with BPD and SAD, and replication with more diverse samples is needed. Moreover, the current study used a between-groups design based on a categorical conceptualization of BPD and SAD, but both BPD symptoms and social anxiety can be considered on a continuum [e.g., 69, 70]. Future studies assessing BPD and social anxiety in a dimensional fashion could help elucidate whether negativity in impression formation increases depending on the level or BPD or SAD pathology. Studies should aim to more thoroughly characterize what this increase looks like (e.g., is it a linear increase) and how the two pathologies may interact.

Second, the adjectives we selected represent a limited set of dimensions and the effects we observed may not hold when asking individuals with BPD or SAD to evaluate targets on other dimensions. The items we chose were meant to capture traits that are important for approach behavior and the forming of social bonds and to reflect whether another person is seen as likely to be a good and fair interaction partner. Other dimensions, e.g., intelligence or conscientiousness, may be less thematically relevant for BPD or SAD and might not result in the same pattern of unfavorable ratings that we observed. At the same time, there are likely also dimensions that are thematically *more* relevant to BPD and SAD than those used herein. For instance, dimensions pertaining to an expectation of being ridiculed or judged by someone (e.g., ‘judgmental’, or ‘deprecatory’), may be relevant to both BPD and SAD. Other dimensions may be more relevant to one group than the other. For instance, qualities signaling distance (e.g., ‘distanced’, ‘detached’) could be experienced as more aversive by BPD than by SAD individuals due to fears of abandonment in BPD individuals that may not be present for individuals with SAD. At the same time, dimensions signaling that someone is in a superior position (e.g., ‘superior’, ‘powerful’, ‘in charge’, ‘competent’), which tend to be associated with evaluative contexts, may be more relevant to SAD than to BPD individuals. Thus, future work should aim to carefully select rating dimensions, assess their relevance for specific participant groups, and further determine whether the effects are restricted to specific dimensions in impression formation.

A further limitation is that, due to the relative novelty of the mouse-tracking method, no universally agreed upon standard has yet been established for designing and analysing mouse-tracking studies. As such, we made a number of decisions when designing the current mouse-tracking study. In particular, we chose a commonly employed mouse-tracking setup in which participants do not receive specific instructions regarding how to move the mouse (as opposed to explicitly instructing them or forcing them to start moving early) and in which participants can indicate their choice via clicking a button (instead of simply touching it without a click). The advantage of this setup was that it is very easy for participants to familiarize themselves with it, which we deemed important when extending mouse-tracking methodology to clinical samples, which are likely less familiar with the complex technical setups that are often used in frequently-studied populations of psychology students. At the same time, other setups, especially those that enforce early movements of participants, have been shown to lead to larger effects in the mouse trajectories [see 71]. Thus, future studies could consider modifying the methodological setup of the current study. Concerning the analysis of mouse trajectories, it may be important to consider the complete shape of mouse trajectories instead of relying only on numeric summary statistics like MAD values [52]. Because of this, we replicated all our analyses by classifying the individual trajectory shapes into prototypes and found that the findings of the MAD analyses replicated.

### Implications

The present evidence suggests that women with BPD and SAD tend to evaluate strangers more unfavorably than HCs, though the SAD group differed from HCs only in their agreement with negative adjectives. Nonetheless, unfavorable evaluations largely reflected a tendency to reject positive attributes, for instance evaluating others as ‘not humble’, rather than a tendency to ascribe negative attributes to targets, such as ‘greedy’. Even though BPD and SAD individuals showed higher rates of agreeing with negative adjectives than HCs, the overall frequency of agreeing with negative adjectives was less than 20%. Therefore, cognitive strategies that train individuals to focus on positive qualities in others could be more effective for remedying interpersonal problems and loneliness characteristic of BPD and SAD populations than focusing on re-appraising the ascription of negative traits, as this tended to be rare.

Process-level analyses using mouse-tracking revealed that women with BPD and SAD experienced a comparable amount of cognitive conflict when rejecting as compared to agreeing with positive attributes, whereas healthy women experienced more conflict when rejecting them. It is possible that this reflects differences in the learning history between those with and without psychopathology, such that BPD and SAD individuals have learned from experience to expect fewer good things from others than HCs. The high rates of interpersonal trauma and invalidating family environments that have been reported for BPD could support this notion [e.g., 68, 72] as could the ample evidence of negative reactions from others that are reported for the SAD population [e.g., 73–75].

At the same time, all rater groups experienced more cognitive conflict when actively ascribing a negative quality to someone than when rejecting it. Thus, even though they eventually made more unfavorable evaluations such as ‘the person is greedy’ as compared to HCs, BPD and SAD individuals also appeared to consider the positive option (‘not greedy’) during their decision process. This implies that agreement with negative adjectives was not an intuitive or automatic response pattern, but that BPD and SAD individuals actively discarded the positive option before coming to their unfavorable evaluation. This holds opportunity for implementing strategies rooted in cognitive behavioral therapy that build on the existing activation of the positive response option and strengthen its relative value. These could be well integrated into existing approaches that already emphasize interpersonal skills, such as dialectical behavior therapy [15]. A second treatment avenue could be to use mentalization based approaches [29] that help participants remain open-minded toward their interaction partner so as not to form premature impressions. Of course, replication of the present findings is necessary before any specific interventions should be derived.

Finally, the inclusion of both positive and negative adjectives revealed a number of important findings that would have been overlooked if the material had not included stimuli of both valences. Thus, an inclusion of positive and negative material appears vital to further assess impression formation in future studies. In addition to including both valences, a careful selection of rating dimensions with varying relevance to different disorders is also necessary to further determine where patient groups may systematically converge or differ in their assessment of strangers. Future studies should consider sampling participants with a range of psychopathology in a dimensional fashion to further assess the potential transdiagnostic nature of negative impression formation.

## Supporting information

S1 Appendix (DOCX)

S1 Text (DOCX)

## Abbreviations

BPD: Borderline Personality Disorder
SAD: Social Anxiety Disorder
HC: Healthy Control participants
DD: Depression /dysthymia
MAD: Maximum absolute deviation
