# Supplementary material for: Mouse-tracking reveals cognitive conflict during negative impression formation in women with Borderline Personality Disorder or Social Anxiety Disorder
Source: PLoS One. 2021 Mar 4;16(3):e0247955. doi: 10.1371/journal.pone.0247955 (PMC7932102; doi:10.1371/journal.pone.0247955)
Supplement: S1 Appendix — (DOCX) [file pone.0247955.s001.docx]

**S1 Appendix.**

It was recently argued that mouse-tracking studies should go beyond analyzing trial summary statistics such as MADs and instead take into account the complete shape of the trajectory (52). We therefore performed additional analyses for hypothesis 2, using the classified trajectory type instead as an outcome. The idea of this novel classification procedure is that trajectories can be assigned to one of several, a priori specified prototype trajectories from which they have the smallest Euclidean distance. We used a set of five prototype trajectories (see Fig A1) based on a meta-analysis by Wulff, Haslbeck (52) that describe the majority of observed trajectories in different methodological settings (71).

These prototypes include straight trajectories from the start to the response button, curved trajectories that slightly deviate towards the unchosen alternative, and three types of change of mind trajectories. Continuous change of mind trajectories (cCoM) are curved trajectories with a strong deviation towards the non-chosen option. In discrete change of mind trajectories (dCoM), participants move their cursor all the way into the response box of the unchosen option and then back to the other side of the screen. In double discrete change of mind trajectories (dCoM2), participants first move towards the ultimately chosen option, from there horizontally to the non-chosen option and then back to the chosen option. Using the individual trajectory classifications, we performed a mixed ordinal regression using the clmm function from the ordinal package in R. We entered the five prototypes in ascending order of cognitive conflict (straight < curved < cCoM < dCoM < dCoM2) as an ordinal outcome and predicted it with the BPD and SAD dummies and their interaction, adjusting for the DD dummy and coding whether participants made a negative evaluation or not. Results are presented in Table A1 and show that all MAD-based findings replicated using the prototype approach.

**Fig A1. Schematic depiction of prototypes used for the supplemental analyses.**


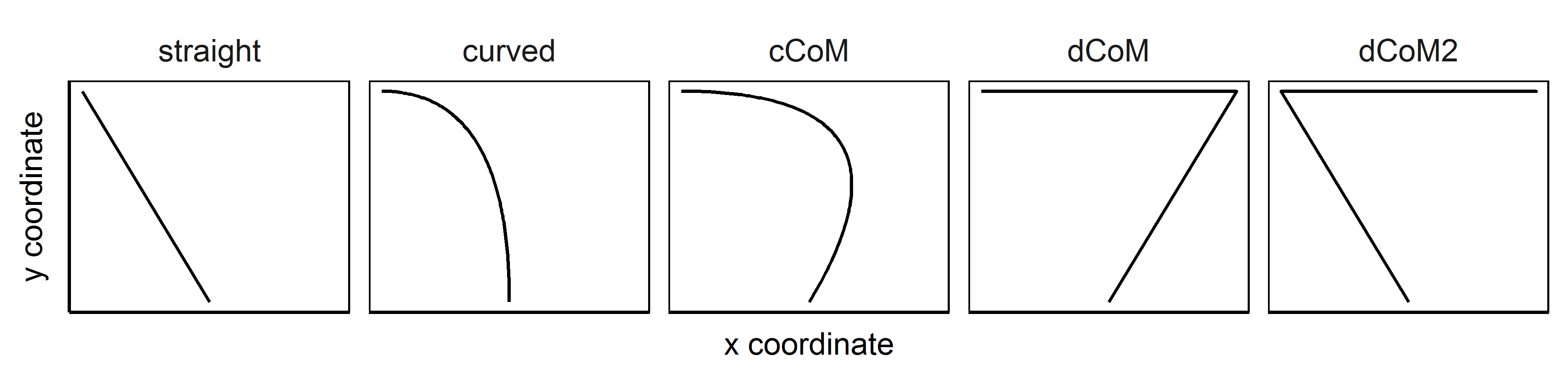


**Table A1. Results from two ordinal mixed effects models with prototypes for mouse-tracking trajectories as outcomes, predicted by diagnostic group and decision type, presented separately for negative and positive adjectives and for each of the rater group baselines.**

|  | *Positive adjectives* | | |  | *Negative adjectives* | | |
| --- | --- | --- | --- | --- | --- | --- | --- |
| *Predictor* | *Est.* | *SE* | *p* |  | *Est.* | *SE* | *p* |
|  | *HC Rater Baseline* | | | | | | |
| Positive evaluation | **-0.21** | **0.05** | **<.001** |  | **-1.02** | **0.09** | **<.001** |
| BPD (vs. HC) | -0.17 | 0.22 | .453 |  | -0.07 | 0.24 | .777 |
| SAD (vs. HC) | -0.20 | 0.21 | .332 |  | -0.29 | 0.23 | .197 |
| DD covariate | 0.06 | 0.23 | .779 |  | 0.12 | 0.24 | .619 |
| Positive evaluation×BPD | **0.27** | **0.07** | **<.001** |  | 0.01 | 0.12 | .950 |
| Positive evaluation×SAD | **0.15** | **0.07** | **.022** |  | **0.29** | **0.12** | **.018** |
|  | *BPD Rater Baseline* | | | | | | |
| Positive evaluation | 0.06 | 0.05 | .215 |  | **-1.01** | **0.08** | **<.001** |
| HC (vs. BPD) | 0.17 | 0.22 | .453 |  | 0.07 | 0.24 | .777 |
| SAD (vs. BPD) | -0.04 | 0.21 | .871 |  | -0.22 | 0.23 | .339 |
| DD covariate | 0.06 | 0.23 | .779 |  | 0.12 | 0.24 | .619 |
| Positive evaluation×HC | **-0.27** | **0.07** | **<.001** |  | -0.01 | 0.12 | .949 |
| Positive evaluation×SAD | -0.12 | 0.07 | .085 |  | **0.28** | **0.11** | **.012** |
|  | *SAD Rater Baseline* | | | | | | |
| Positive evaluation | -0.06 | 0.05 | .236 |  | **-0.73** | **0.08** | **<.001** |
| HC (vs. SAD) | 0.20 | 0.21 | .332 |  | 0.29 | 0.23 | .197 |
| BPD (vs. SAD) | 0.04 | 0.21 | .871 |  | 0.22 | 0.23 | .339 |
| DD covariate | 0.06 | 0.23 | .779 |  | 0.12 | 0.24 | .619 |
| Positive evaluation×HC | **-0.15** | **0.07** | **.022** |  | -0.29 | 0.12 | .018 |
| Positive evaluation×BPD | 0.12 | 0.07 | .085 |  | **-0.28** | **0.11** | **.013** |

*Note.* BPD = borderline personality disorder, SAD = social anxiety disorder / anxious avoidant personality disorder, HC = healthy control participants, DD = current major depressive episode or dysthymia. Positive evaluation coded 0.5 when raters evaluated targets positively and -0.5 when they evaluated targets negatively. Significant effects are highlighted in boldface.
