# Supplementary material for: Mouse-tracking reveals cognitive conflict during negative impression formation in women with Borderline Personality Disorder or Social Anxiety Disorder
Source: PLoS One. 2021 Mar 4;16(3):e0247955. doi: 10.1371/journal.pone.0247955 (PMC7932102; doi:10.1371/journal.pone.0247955)
Supplement: S1 Text — (DOCX) [file pone.0247955.s002.docx]

**S1 Text.**

We note that there are three previous publications from the same study program that are related to the manuscript we are submitting. All three articles were published in peer-reviewed journals:

Hepp, J., Störkel, L. M., Kieslich, P. J., Schmahl, C., & Niedtfeld, I. (2018). Negative evaluation of individuals with borderline personality disorder at zero acquaintance. *Behaviour Research and Therapy*, 111, 84-91. https://doi.org/10.1016/j.brat.2018.09.009

Hepp, J., Gebhardt, S., Kieslich, P. J., Störkel, L. M., & Niedtfeld, I. (2019). Low positive affect display mediates the association between borderline personality disorder and negative evaluations at zero acquaintance. *Borderline Personality Disorder and Emotion Dysregulation, 6*(1), 4. https://doi.org/10.1186/s40479-019-0103-6

Hepp, J., Kieslich, P. J., Schmitz, M., Schmahl, C., & Niedtfeld, I. (2020). Negativity on two sides: Individuals with Borderline Personality Disorder form negative first impressions of others and are perceived negatively by them. *Personality Disorders: Theory, Research, and Treatment, Online publication ahead of print*. https://doi.org/10.1037/per0000412

In Hepp et al. (2018) we first described the target sample consisting of 26 individuals with BPD and 26 healthy control participants. Target participants were filmed while speaking about their personal preferences and later functioned as stimuli in the Thin Slices paradigm. Within the same publication, we reported data from two samples of students, who we showed these target videos to. The focus of the investigation was to assess whether students would evaluate targets differently depending on whether the target was in the BPD or the HC group. Hence, the focus of the study were *target* *effects* and not rater effects as in the present study. The two rater samples reported in Hepp et al. (2018) have no overlap with the rater sample we report in the present study. Both studies used the same targets for the Thin Slices paradigm.

In Hepp et al. (2019) we showed the target videos to a new sample of student raters and asked them to rate observable behaviors (amount of eye contact, positive and negative facial affect expression). Again, the focus was on whether the two target groups differed on these dimensions. The rater sample reported in Hepp et al. (2019) has no overlap with the rater sample we report in the present study. Both studies used the same targets for the Thin Slices paradigm.

In Hepp et al. (2020) we reported a different subset of data from the same rater sample that is also included in the present study (the BPD, SAD, and HC raters). Raters again saw the Thin Slices target videos and evaluated these. First, raters evaluated targets based on their performance in an economic game and provided trustworthiness, approachability, and similarity ratings. None of these outcomes were mouse-tracked and the results for these variables are reported in Hepp et al. (2020). The study addressed interaction effects between target and rater groups (e.g., do BPD raters perceive BPD targets differently). In the current study, we report results for the mouse-tracking trials that raters completed (evaluation of targets based on positive and negative adjectives). These variables are reported here for the first time. Direct overlap between Hepp et al. (2020) and the current study is only in the demographic data for the rater sample. There is no overlap in the outcomes we reported in Hepp et al. (2020) and the present study.
